# Phase Transition Effect on Ferroelectric Domain Surface Charge Dynamics in BaTiO_3_ Single Crystal

**DOI:** 10.3390/ma14164463

**Published:** 2021-08-09

**Authors:** Dongyu He, Xiujian Tang, Yuxin Liu, Jian Liu, Wenbo Du, Pengfei He, Haidou Wang

**Affiliations:** 1 National Key Laboratory for Remanufacturing, Army Academy of Armored Forces, Beijing 100072, China; xiujtang350@126.com (X.T.); lyxinbd@163.com (Y.L.); mengly0603@163.com (W.D.); hepengfei93@163.com (P.H.); wanghaidou@tsinghua.org.cn (H.W.); 2 National Engineering Research Center for Remanufacturing, Army Academy of Armored Forces, Beijing 100072, China; xsliu218@126.com; 3 Advanced Interdisciplinary Technology Research Center, National Innovation Institute of Defense Technology, Beijing 100071, China

**Keywords:** ferroelectric domain, domain wall, surface charge, phase transition

## Abstract

The ferroelectric domain surface charge dynamics after a cubic-to-tetragonal phase transition on the BaTiO_3_ single crystal (001) surface was directly measured through scanning probe microscopy. The captured surface potential distribution shows significant changes: the domain structures formed rapidly, but the surface potential on polarized ***c*** domain was unstable and reversed its sign after lengthy lapse; the high broad potential barrier burst at the corrugated ***a-c*** domain wall and continued to dissipate thereafter. The generation of polarization charges and the migration of surface screening charges in the surrounding environment take the main responsibility in the experiment. Furthermore, the ***a*-*c*** domain wall suffers large topological defects and polarity variation, resulting in domain wall broadening and stress changes. Thus, the ***a-c*** domain wall has excess energy and polarization change is inclined to assemble on it. The potential barrier decay with time after exposing to the surrounding environment also gave proof of the surface screening charge migration at surface. Thus, both domain and domain wall characteristics should be taken into account in ferroelectric application.

## 1. Introduction

The development of ferroelectric materials has promoted promising applications in non-volatile storage devices [1,2,3], sensors and actuators [4,5], and infrared detector systems [6,7]. The ferroelectric domain structure is the fundamental execution unit of ferroelectrics [8]. Below the Curie temperature [9], ferroelectrics have spontaneously polarized domains and their ability to switch with the external field is the most important feature in ferroelectric physics [10,11]. Surface charges can reflect the underlying domain polarity characteristics [12]. Thus, temperature and surface charges affect the polarization dynamics and domain structures. Studying the change of ferroelectric domain performance with temperature has great scientific and engineering significance, for as electronic components, ferroelectrics always work in a certain temperature field environment [13,14,15].

To figure out the nature of ferroelectric properties, it is necessary to perform nanoscale or microscale imaging of the local domain structure. Such studies should pay special attention to domains and domain walls, for their static and dynamic behaviors determine the stability of ferroelectrics [16,17]. Thus, it is important to use direct imaging techniques to study the characteristics of domain structure [18,19,20]. Scanning probe microscopy (SPM), which allows the high-resolution and nondestructive imaging for domain structures and observation of their dynamic behaviors on the micro and nanoscale, has been used [21,22,23,24]. The research of ferroelectric surface science using SPM has provided deep insight into the structure and dynamics of ferroelectrics [25,26,27]. Among the various SPM techniques, the Kelvin probe SPM (SPKFM: Scanning Kelvin Probe Force Microscopy) can reflect the surface electrostatic forces, and is used to visualize and detect the distribution and the dynamics of the polarized charges on ferroelectric surfaces [28,29]. As such, during the experiment, the SKPFM was used to measure the surface charge dynamics of the ferroelectric domain in situ. Based on converse piezoelectric effect, the piezoelectric response force microscopy (PFM: Piezo-response force microscope) utilizes a conductive probe to detect the local deformation of the sample in response to an external excitation voltage applied to the tip, thereby providing valuable information of ferroelectric domain structures [30]. Thus, in the experiments, PFM was also introduced to characterize the domain structure and the surface charge dynamics [31].

Compared with other ferroelectrics, BaTiO_3_ has a lower Curie temperature of 120 °C [32], which is suitable and convenient for the study domain evolution during phase transition in the actual experimental process [13,14]. In this article, we introduced the SKPFM study of domain structures and dynamic behaviors of BaTiO_3_ single crystal after a cubic-to-tetragonal phase transition. In the experiments, the ***c*** domain surface potential reversal and high broad potential barriers at ***a-c*** domain walls were observed. This phenomenon was captured around the polarized ***c*** domains, whose spontaneous polarization vector points upward or downward to the (001) surface [13]. Thus, the ***c*** domain spontaneous polarization charge assembled at the top (001) surface immediately after the phase transition; in the meantime, the surface screening charge moved to the surface to compensate the charged surface. The decay of the potential barrier confirms the surface screening charge migration. The topological defect around the ***a-c*** domain wall caused the boundary to widen and the stresses/strains to change, which provided excess energy for capturing surface charges. Therefore, polarization charges and the surface charges should be considered fundamentally in ferroelectric research and application.

## 2. Materials and Methods

A single crystal of (001) orientation BaTiO_3_ with dimension of 5 × 5 × 1 mm^3^ was used during the experiment. The (001) surface was mechanically ground using sandpaper and polished using diamond lapping pastes and silica colloidal suspension (0.05 mm), whereby surface roughness of less than 3 nm was achieved. Then, the sample was cleaned by ultrasonication in deionized water for 100 s. After that, the sample was placed on a heating plate and the temperatures were controlled by heating the plate during the experiments. The Lakeshore 335 Temperature Controller was utilized to drive the heating plate, and the temperature of the heating plate can be controlled from ambient to 250 °C with an accuracy of ±0.5 °C. In the experiment, the heating rate was maintained at 100 °C per minute.

A Bruker Nanoscope V SPM system (Dimension V, Bruker, Santa Barbara, CA, USA) was used to characterize the domain structure and dynamics of BaTiO_3_ single crystal surface with a conductive tip (NSG01/W_2_C from NT-MDT, W_2_C-coated) with a resonance frequency of 150 kHz in ambient conditions. Surface potential measurements (SKPFM) are based on tapping mode, and the tip was operated in a 100 nm tip-surface lift height with an AC voltage of 3 V amplitude at a frequency of 135 kHz. The scan rate was set to 0.8 Hz at 512 lines per frame. Thus, it took approximately 12 min to complete a whole image scanning.

In this work, to measure the surface charge dynamics of BaTiO_3_ domain structure after a cubic-to-tetragonal phase transition, the sample was treated as follows: the crystal was placed in ambient at room temperature (RT) at first, then heated from RT to 135 °C (above the *T_C_* at 120 °C). The crystal was kept at 135 °C for 30 min for equilibration, and then cooled to 90 °C at a rate of 100 °C/min. In other words, the crystal structure underwent a process of changing from the tetragonal to cubic phase and then cooling to the tetragonal phase. Finally, new domain structures were formed in the crystal. In the meantime, the SKPFM was used to detect the domain structure and surface charge dynamics by surface potential and AFM topography signals.

## 3. Results and Discussion

**Figure 1:**
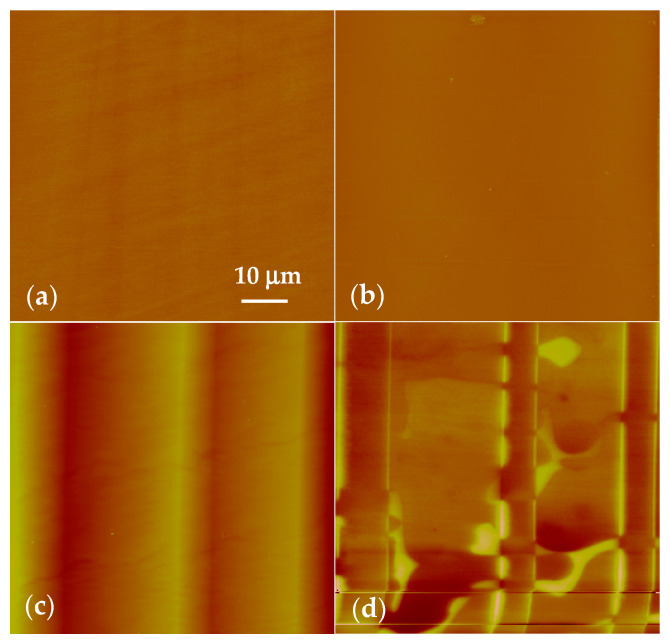
Phase transitions from cubic (135 °C) to tetragonal (90 °C). AFM topography (**a**) and surface potential map (**b**) of the (001) BaTiO_3_ surface acquiring at 135 °C which is above the Curie temperature of BaTiO_3_ at 120 °C with a 40 nm Z scale and the roughness was 0.68 nm. AFM topography image with a 200 nm Z scale (**c**) and surface potential mapping with a 0.6 V Z scale (**d**) acquired after cooling to 90 °C showing typical ***a-c*** domain structures.

The phase transition of BaTiO_3_ crystal leads to the surface morphology and surface charge distribution change that observed in the SPM images (Figure 1). Figure 1a displays the surface topography of the sample with a 40 nm Z scale at 135 °C. Above *T_C_*, BaTiO_3_ single crystal was in cubic phase and there is no spontaneously polarized domain inside the crystal. No fluctuation and charge were on the surface. Thus, no domain contrast was observed in the topography image above the *T_C_*. Meanwhile, the surface potential distribution was zero, as shown in Figure 1b. After cooling to 90 °C, the crystal was in tetragonal phase and domain structures were formed in the sample. Each domain polarization can be distinguished, and the distinct changes of both the ferroelectric domain pattern and the surface charge were observed. The corrugated topography image acquired at 90 °C show the 90° ***a-c*** domain structure, as marked in Figure 1c. The straight vertical line was attributed to the 90° ***a-c*** domain wall, which was the surface topological defect caused by the lattice parameters change on each side, showing the change in structure integrity and the asymmetry of lattice mismatch. The surface potential distribution of the sample changed significantly, as shown in Figure 1d. The potential distribution with distinct contrast presented the characteristics of bright, dark, and intermediate. For BaTiO_3_, the polarization vector in the ***c*** domain points either downward to the bottom or upward to the top (001) surface, producing a negative (***c*^−^** domain) or positive (***c*^+^** domain) charge on the surface. In the SKPFM images, dark and bright contrasts correspond to the negative and positive regions, respectively. Thus, the region displayed as dark is attributed to the ***c*^−^** domain, and the region displayed as bright is attributed to the ***c*^+^** domain. The ***c*^+^** and ***c*^−^** domain walls are curved in the surface potential image (0.6 V Z scale) with no topological mismatch at the boundary. The measured surface potential value of the ***c*^+^** and ***c*^−^** domains are +180 mV and −250 mV, respectively. The ***a*** domain is parallel to (001) surface, thus, no charge generates on the surface; the surface potential is zero, showing the intermediate contrast in between the bright (***c*^+^** domain) and dark (***c*^−^** domain) areas, as shown in Figure 1d. In addition, the surface potential features changed significantly not only in the domains, but also at the domain wall regions. Bright and dark potential barriers connected to the adjacent ***c*^−^** and ***c*^+^** domains burst at the ***a-c*** domain wall area in Figure 1d. The potential height at the domain wall can reach the value of 300 mV, and the domain wall width was up to 2 μm, broader than the domain wall polarization [21].

**Figure 2:**
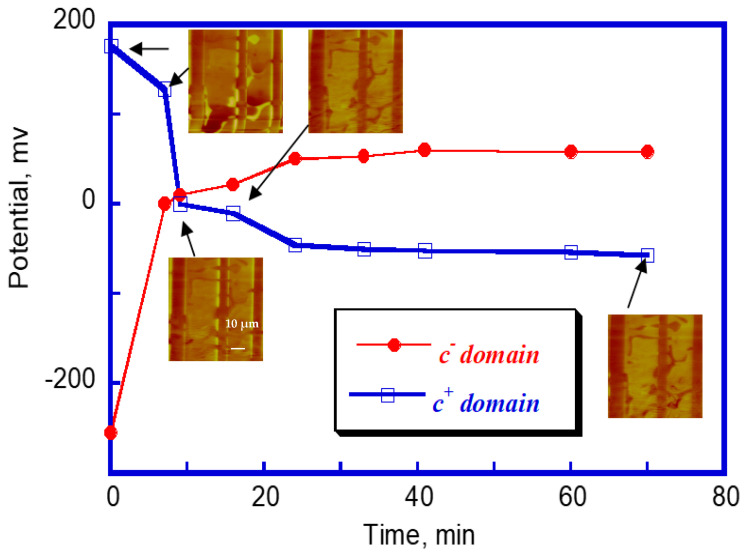
The ***c*^+^** and ***c*^−^** domain potential versus time showing an inversion of the potential sign, the inserted images are subsequent scans of the same area showing domain potential reversal and the decay of potential barrier. (The arrows point to the initial ***c*^+^** domain data points.)

**Figure 3:**
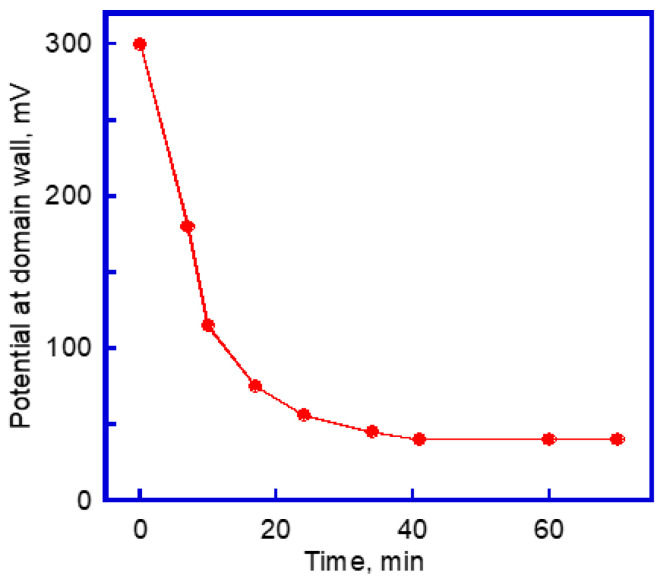
The intensity of 90° ***a-c*** domain wall potential change over time.

The captured surface potential distribution was unstable and changed fast during the mapping (Figure 2). The ***c*** domain surface potential inversed its sign with scanning; that is, the original bright ***c*^+^** domains apparently became negative, and the dark negative ***c*^−^** domains turned into positive stripes. The dark regions completely replaced the bright regions. The domain potential reversal was quite fast, which was observed only during the first 10 min. The high potential stripes were acquired at the 90° ***a-c*** domain wall, but gradually decayed with time with both the height and the width continued to decrease. The relaxation of the high potential barrier decayed quite fast during the initial 20 min, and then slowed down, as shown in Figure 3. After 70 min, the domain and domain wall surface potential equilibrium state were achieved. The domain structure with the bright, dark and intermediate regions was obtained, and corresponded to the initial ***c*^−^**, ***c^+^*** and ***a*** domain areas, respectively. The overall change in the surface potential dynamics is summarized in Figure 2.

The simultaneous observation of domain surface potential reversal and the domain wall potential barriers has not yet been reported. The newly formed ***c*** domain surface potential was unstable and has reversed its sign. High broad potential barriers burst around the ***a-c*** domain wall, and gradually decayed with exposure to the surrounding environment. The surface charge dynamics are associated with the polarized ***c*** domain regions. For BaTiO_3_ single crystal, the ***c*** domain surface gathers the polarization charges [10]. Thus, the observed surface potential instability may be attributed to surface polarization charges motion.

When the ferroelectrics are below the Curie temperature, domains would maintain spontaneous polarization and generate charges on the surface [8]. Initially, the ferroelectric surface is in a completely unscreened state [29,33], and the surface potential image reflects the intrinsic polarization characteristics of the ferroelectric domain structures. The surface potential of ferroelectric domains in an unscreened state is determined by the characteristic of surface polarization charges σ, given by σ = P · *n*, where P is the polarization vector and *n* is the unit perpendicular to the top surface [9]. The energy of the unscreened surface is theoretically 3.49 eV [34]; thus, polar molecules, free electrons or space charges, collectively referred to as screening electronics, tend to be adsorbed onto the polar ***c*** domain surface to neutralize the high energy [13]. With the presence of screening species, the surface screening charge density increases until the polarization charges are completely screened [35]. Thus, the ultimate polarization state of the ferroelectric surface after a long-term lapse depends on the interaction between the spontaneous polarization and the screening charge with the opposite sign [36]. As such, the sign of the surface potential measured by SKPFM is eventually reversed. The high broad potential barrier at the corrugated ***a-c*** domain wall indicates the presence of charges and provides a model of charge accumulation to the wall [13]. The decay of the surface barrier also clarifies the characteristics of surface charge migration [37]. Therefore, the role and properties of domain walls cannot be ignored in ferroelectric performance [38,39,40,41].

**Figure 4:**
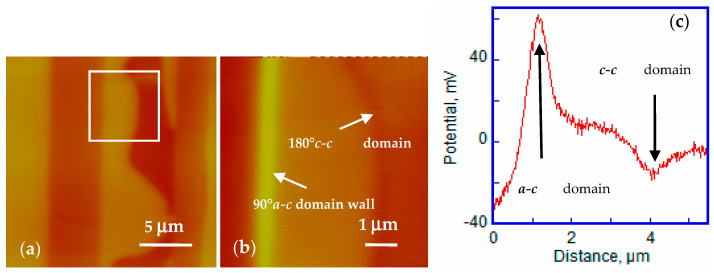
(**a**) Topography map of large-scale ***a-c*** domain structure. The square marked in the image is the zoom-in area used for domain wall potential barrier observation. (**b**) Surface potential image of the zoom in area acquired after cooling showing high broad potential barriers around ***a-c*** domain and ***c-c*** domain wall. (**c**) The surface potential cross section profile showing the width and height of the potential barriers.

To further understand the local domain wall surface potential distribution, a selected area SKPFM variable temperature measurement was carried out, including a large-scale scan (22 μm × 22 μm) and a selected zoom-in area (6 μm × 6 μm). Figure 4a shows the surface potential map of the large-scale domains at 80 °C. The square in Figure 4a is the selected zoom-in area showing a typical ***a-c*** domain structure with the bright, dark and intermediate contrast on the surface potential image. Then, after cooling to 60 °C, the surface potential map of the selected zoom in area was captured immediately (Figure 4b). Potential barriers burst at both the 90° ***a-c*** domain wall and the 180° ***c-c*** domain wall. A cross-section profile was drawn to characterize the potential barrier location and height in Figure 4c. The potential barrier aligns with the 90° ***a-c*** domain wall and the 180° ***c-c*** domain wall; in addition, the height of 90° ***a-c*** domain wall was much higher compared with that of 180° ***c-c*** domain wall. Thus, it indicates that the corrugated 90° ***a-c*** domain wall is inclined to attract more charge to the surface, which seems to be a natural trap for charges. Meanwhile, the observed potential barriers decay gradually at 60 °C.

**Figure 5:**
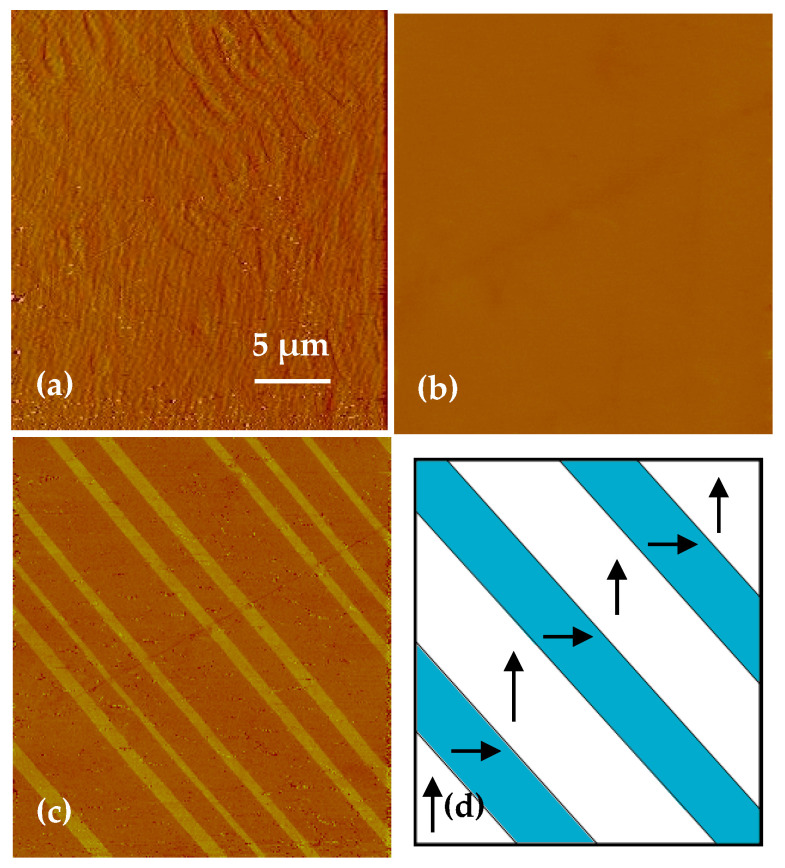
(**a**) AFM topography map of the 90° ***a-b*** domain structure at the (001) plane with a 40 nm Z scale and the roughness was 1.07 nm. (**b**) Surface potential map of the observed 90° ***a-b*** domain zone showing no contrast and no charge. (**c**) In-plane PFM map of the same area showing a typical 90° ***a-b*** domain structure. (**d**) Schematic of the measured ***a-b*** domain zone connected head to tail and perpendicular to each other.

**Figure 6:**
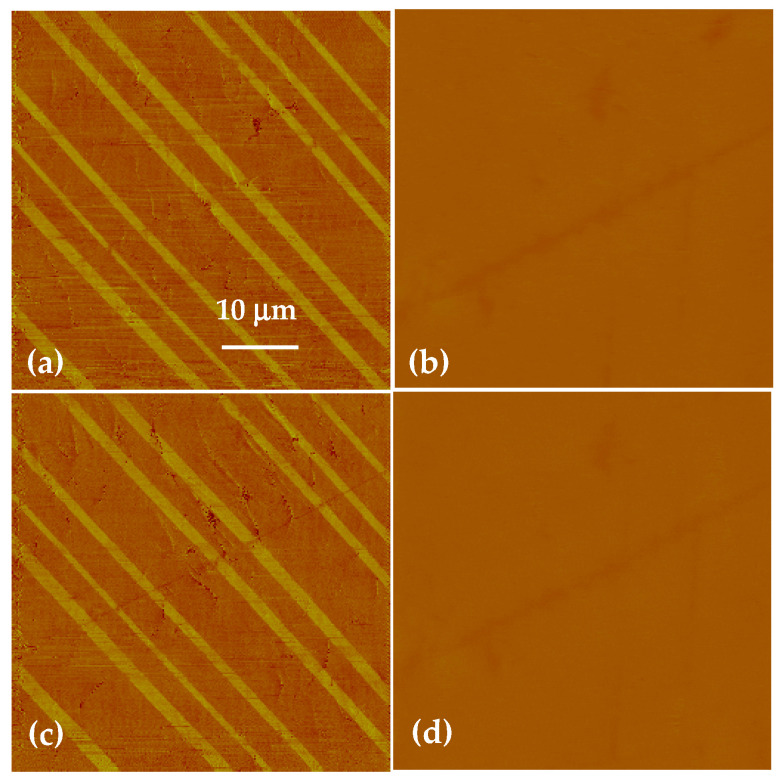
The PFM (**a**) and the corresponding surface potential map (**b**) of 90° ***a-b*** domain at 110 °C, and PFM (**c**) and surface potential image (**d**) after cooling to 90 °C.

To fully analyze the local surface charge effects, the surface dynamics of ferroelectric ***a*** domain with temperature various was also studied. The 90° ***a-b*** domain structures are composed of the ***a*** domain with its polarization vector parallel to the (100) direction and the ***b*** domain with its polarization vector parallel to the (010) direction. Thus, the polar charges only accumulated on the (100) and (010) surfaces, and no polarization charge generates at the observed (001) surface. The polarization vectors of the ***a*** domains and ***b*** domains are connected end-to-end and perpendicular to each other to minimize the system energy, as shown in the schematic of Figure 5d. The surface topography and potential distribution of the 90° ***a-b*** domains on the BaTiO_3_ (001) surface are shown in Figure 5a,b, respectively. The roughness of the surface was measured to be 1.07 nm. It can be determined that the ***a-b*** domain structure cannot be distinguished with no contrast and no corrugation at the surface. Furthermore, the PFM was introduced to characterize the ***a-b*** domain structure and the surface charge dynamics. In the experiments, a 10 V AC signal with a 15 kHz frequency was applied to the tip to induce surface displacement. The ***a-b*** domain structure could be clearly distinguished from the PFM image in Figure 5c. The sample was heated to 110 °C at 5 °C/min and kept for 30 min to reach equilibrium, after which PFM images and surface potential images were captured, as shown in Figure 6a,b. After cooling to 90 °C at 100 °C/min, PFM images and the surface potential images were acquired in situ, as seen in Figure 6c,d. No obvious change in surface appearance or surface charge was observed. In other words, the in-plane polarized ***a-b*** domain structure has no potential on the (001) surface. Thus, the potential barrier induced by thermal variation has a significant relationship with the surface polarization charges. Only when there are polarization charges on the surface will potential barriers appear at domain walls.

As the temperature changes, high and broad potential barriers were observed at the ***a-c*** domain wall and the ***c-c*** domain wall but not at the ***a-b*** domain wall. The charge accumulation at the domain walls around the ***c*** domains may be related to the asymmetry of the charge distribution around the domain wall [42]. The discovery of enhanced local conductivity at ferroelectric domain walls was also reported [43,44]. The domain wall acts as a trap or a path for charges [13]. The polarized ***c*** domain surface charge is inclined to accumulate near the ***c*^+^*-c*^−^** domain walls and ***a-c*** domain walls. Meanwhile, the ***a-b*** domain wall has no polarization charge on the surface and no charge traps.

In addition, domain walls show large topological asymmetry. The ***a-c*** domain walls are connected by domains with a 90° orientation, resulting in lattice mismatch. Thus, the domain wall exhibits a corrugated angle [45]. Separating domains with opposite polarizations, the ***c-c*** domain wall has no lattice mismatch [46]. Studies have also found that the 90° ***a-c*** domain wall of BaTiO_3_ crystal is wider than the 180° ***c-c*** domain wall [40]. To a certain extent, the broadening of the 90° ***a-c*** domain wall can absorb more charges; on the other hand, it also inhibits the movement of surface charges, resulting in an increase in surface potential [47]. Furthermore, domain walls produce large strains associated with the lattice arrangement. When the polarization directions around the domain wall change abruptly, the inhomogeneous stresses and/or strains emerge at the vicinity of the domain wall [45]. In the 180° ***c-c*** domain wall, the strain transitions smoothly from one spontaneous polarization value to the opposite one. Whereas, in the ***a-c*** domain wall, the tilted domain wall polarization causes large strains to sustain the domain patterns [48]. The broadened and highly stressed ***a-c*** domain wall has different physical properties from the smooth transition ***c-c*** domain wall, resulting in asymmetry in domain wall charge accumulation and distribution. During the thermal variation, the surface polarization charge accumulation occurs with the change of surface free energy $\Delta E=\left(E_{wall}+E_{charge}-E_{wall,charge}\right)>0$. Compared with the ***c-c*** domain wall, the ***a-c*** domain wall has instantaneous excess free energy [49]. Thus, the ***a-c*** domain wall can attract more surface charges, resulting in a high potential barrier around it. As the charged surface is exposed to the surrounding environment, the screening species migrates onto the surface to neutralize the surface charges, leading the high potential barriers’ decay with time [36].

**Figure 7:**
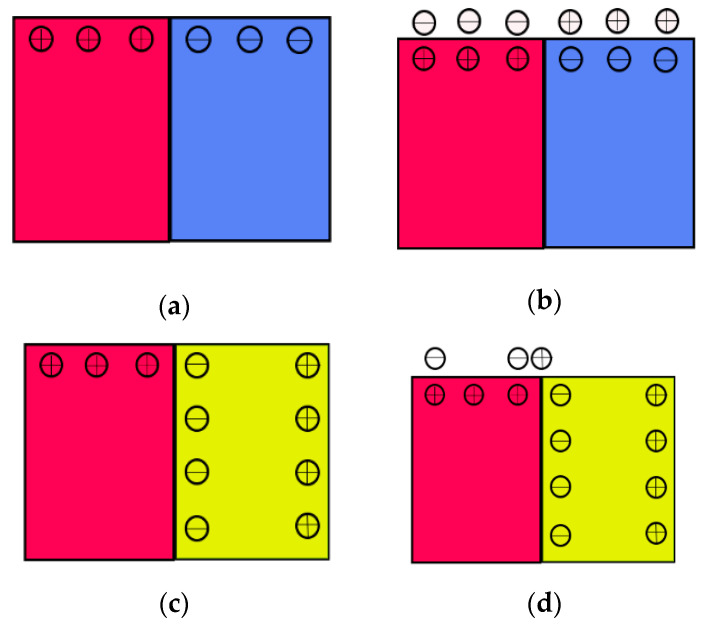

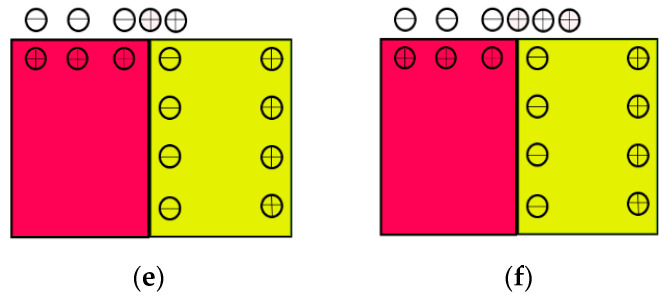
Schematic of surface charge and the screening charge mobility on ferroelectric surface. (**a**,**b**) The surface charge and the screening of c domain surface, (**c**–**f**) the surface charge mobility of ***a-c*** domain.

Thus, after a cubic-to-tetragonal phase transition, the observed ***c*** domain surface potential reversal and the high potential behavior at the domain wall are significantly affected by multiple coupling effects: the dynamics of the ***c*** domain surface potential reversal is attributed to the interaction between polarization charge and surface screening species. The observed potential barrier at the ***a-c*** domain wall is closely associated with the accumulation of the adjacent ***c*** domain polarization charges due to the asymmetry of the physical structure characteristics as well as domain wall topological defects and strain changes (Figure 7).

## 4. Conclusions

In the paper, the ferroelectric domain surface charge dynamics of a (001) BaTiO_3_ single crystal after a cubic-to-tetragonal phase transition were studied using the SKPFM method. A significant change in the BaTiO_3_ domain surface potential after thermal variation was found. The captured ***c*** domains’ surface potential distribution showed a large unstable amplitude and reversed its sign with SKPFM scanning. Bright and dark high potential barriers burst on the corrugated ***a-c*** domain walls, with its sign opposite to the adjacent ***c*** domain, but were not stable and declined continually with time. The surface potential dynamics are relative to that expected from polarization orientation on BaTiO_3_ and the surface screening charge mobility in ambient. The captured potential barrier at the corrugated ***a-c*** domain wall not only depends on the migration charges on polarized ***c*** domain surface, but is also aided by the width of the domain wall broadening and an increase in stress/strain across the wall.

## Data Availability

The raw/processed data required to reproduce these findings cannot be shared at this time as the data also forms part of an ongoing study.

## References

[1] Naber R.C.G., Asadi K., Blom P.W.M., De Leeuw D.M., De Boer B. (2010). Organic Nonvolatile Memory Devices Based on Ferroelectricity. Adv. Mater..

[2] Mikolajick T., Slesazeck S., Mulaosmanovic H., Park M.H., Fichtner S., Lomenzo P.D., Hoffmann M., Schroeder U. (2021). Next generation ferroelectric materials for semiconductor process integration and their applications. J. Appl. Phys..

[3] Park B.H., Kang B.S., Bu S.D., Noh T.W., Lee J., Jo W. (1999). Lanthanum-substituted bismuth titanate for use in non-volatile memories. Nat. Cell Biol..

[4] Martin L.W., Rappe A.M. (2017). Thin-film ferroelectric materials and their applications. Nat. Rev. Mater..

[5] Muralt P. (2000). Ferroelectric thin films for micro-sensors and actuators: A review. J. Micromech. Microeng..

[6] Whatmore R.W., Patel A., Shorrocks N.M., Ainger F.W. (1990). Ferroelectric materials for thermal ir sensors state-of-the-art and perspectives. Ferroelectrics.

[7] Rogalski A. (2012). History of infrared detectors. Opto-Electron. Rev..

[8] Lines M.E., Glass A.M. (2001). Principles and Applications of Ferroelectrics and Related Materials.

[9] Johnson C.J. (1965). Some dielectric and electro-optic properties of batio3 single crystals. Appl. Phys. Lett..

[10] Kalinin S.V., Bonnell D.A., Alvarez T., Lei X., Hu Z., Ferris J.H., Zhang Q., Dunn S. (2002). Atomic Polarization and Local Re-activity on Ferroelectric Surfaces: A New Route toward Complex Nanostructures. Nano Lett..

[11] Nelson C.T., Gao P., Jokisaari J.R., Heikes C., Adamo C., Melville A., Baek S.H., Folkman C.M., Winchester B., Gu Y. (2011). Domain Dynamics during Ferroelectric Switching. Science.

[12] Balke N., Jesse S., Li Q., Maksymovych P., Okatan M., Strelcov E., Tselev A., Kalinin S.V. (2015). Current and surface charge modified hysteresis loops in ferroelectric thin films. J. Appl. Phys..

[13] He D., Xing X., Qiao L., Volinsky A.A. (2014). Temperature change effect on BaTiO_3_ single crystal surface potential around domain walls. Appl. Surf. Sci..

[14] Yuan G., Chen J., Xia H., Liu J., Yin J., Liu Z. (2013). Ferroelectric domain evolution with temperature in BaTiO_3_ film on (001) SrTiO_3_ substrate. Appl. Phys. Lett..

[15] Everhardt A.S., Denneulin T., Grünebohm A., Shao Y.-T., Ondrejkovic P., Zhou S., Domingo N., Catalan G., Hlinka J., Zuo J.-M. (2020). Temperature-independent giant dielectric response in transitional BaTiO_3_ thin films. Appl. Phys. Rev..

[16] Su Y., Landis C.M. (2007). Continuum thermodynamics of ferroelectric domain evolution: Theory, finite element implementation, and application to domain wall pinning. J. Mech. Phys. Solids.

[17] Liu S., Zheng F., Koocher N.Z., Takenaka H., Wang F., Rappe A.M. (2015). Ferroelectric Domain Wall Induced Band Gap Reduction and Charge Separation in Organometal Halide Perovskites. J. Phys. Chem. Lett..

[18] Zheng T., Wu J.G. (2020). Perovskite BiFeO_3_-BaTiO_3_ ferroelectrics: Engineering property by domain evolution and thermal depo-larization modification. Adv. Electron. Mater..

[19] Kalinin S.V., Sumpter B.G., Archibald R.K. (2015). Big-deep-smart data in imaging for guiding materials design. Nat. Mater..

[20] Kim Y., Han H., Lee W., Baik S., Hesse D., Alexe M. (2010). Non-Kolmogorov−Avrami−Ishibashi Switching Dynamics in Na-noscale Ferroelectric Capacitors. Nano Lett..

[21] Matsuura K., Cho Y., Ramesh R. (2003). Observation of domain walls in PbZr_0.2_Ti_0.8_O_3_ thin film using scanning non-linear dielectric microscopy. Appl. Phys. Lett..

[22] Gruverman A., Alexe M., Meier D. (2019). Piezoresponse force microscopy and nanoferroic phenomena. Nat. Commun..

[23] Liu Y., Yu B.X., Liu Z.W., Beck D., Zeng K.Y. (2020). High-Speed Piezoresponse Force Microscopy and Machine Learning Ap-proaches for Dynamic Domain Growth in Ferroelectric Materials. ACS Appl. Mater. Interfaces.

[24] Kwon O., Seol D., Qiao H., Kim Y. (2020). Recent Progress in the Nanoscale Evaluation of Piezoelectric and Ferroelectric Properties via Scanning Probe Microscopy. Adv. Sci..

[25] Lilienblum M., Soergel E. (2011). Anomalous domain inversion in LiNbO_3_ single crystals investigated by scanning probe microscopy. J. Appl. Phys..

[26] Li T., Zeng K. (2018). Probing of Local Multifield Coupling Phenomena of Advanced Materials by Scanning Probe Microscopy Techniques. Adv. Mater..

[27] Kalinin S.V., Jesse S., Tselev A., Baddorf A.P., Balke N. (2015). The Role of Electrochemical Phenomena in Scanning Probe Microscopy of Ferroelectric Thin Films. ACS Nano.

[28] He D.Y., Qiao L.J., Volinsky A.A. (2011). Humidity effect on BaTiO_3_ c-domain surface potential inversion induced by electric field. J. Appl. Phys..

[29] Liu X.Y., Kitamura K.J. (2006). Surface potential imaging of nanoscale LiNbO_3_ domains investigated by electrostatic force microscopy. Appl. Phys. Lett..

[30] Lei S., Eliseev E., Morozovska A.N., Haislmaier R.C., Lummen T.T.A., Cao W., Kalinin S., Gopalan V. (2012). Origin of piezoelectric response under a biased scanning probe microscopy tip across a 180 ferroelectric domain wall. Phys. Rev. B.

[31] Kelley K.P., Ren Y., Morozovska A.N., Eliseev E.A., Ehara Y., Funakubo H., Giamarchi T., Balke N., Vasudevan R.K., Cao Y. (2020). Dynamic Manipulation in Piezoresponse Force Microscopy: Creating Nonequilibrium Phases with Large Electromechanical Response. ACS Nano.

[32] Sakayori K., Matsui Y., Abe H., Nakamura E., Kenmoku M., Hara T., Ishikawa D., Kokubu A., Hirota K., Ikeda T. (1995). Curie temperature of BaTiO_3_. Jpn. J. Appl. Phys..

[33] Schoenherr P., Shapovalov K., Schaab J., Yan Z., Bourret E.D., Hentschel M., Stengel M., Fiebig M., Cano A., Meier D. (2019). Observation of Uncompensated Bound Charges at Improper Ferroelectric Domain Walls. Nano Lett..

[34] Li X., Wang B., Zhang T.-Y., Su Y. (2014). Water Adsorption and Dissociation on BaTiO_3_ Single-Crystal Surfaces. J. Phys. Chem. C.

[35] Yamada T., Ito D., Sluka T., Sakata O., Tanaka H., Funakubo H., Namazu T., Wakiya N., Yoshino M., Nagasaki T. (2017). Charge screening strategy for domain pattern control in nano-scale ferroelectric systems. Sci. Rep..

[36] Copie O., Chevalier N., Le Rhun G., Rountree C.L., Martinotti D., Gonzalez S., Mathieu C., Renault O., Barrett N. (2017). Adsorbate Screening of Surface Charge of Microscopic Ferroelectric Domains in Sol–Gel PbZr_0.2_Ti_0.8_O_3_ Thin Films. ACS Appl. Mater. Interfaces.

[37] Nataf G.F., Guennou M., Gregg J.M., Meier D., Hlinka J., Salje E.K.H., Kreisel J. (2020). Domain-wall engineering and topological defects in ferroelectric and ferroelastic materials. Nat. Rev. Phys..

[38] Gu Y., Li M., Morozovska A.N., Wang Y., Eliseev E., Gopalan V., Chen L.-Q. (2014). Flexoelectricity and ferroelectric domain wall structures: Phase-field modeling and DFT calculations. Phys. Rev. B.

[39] Sharma P., Zhang Q., Sando D., Lei C.H., Liu Y., Li J., Nagarajan V., Seidel J. (2017). Nonvolatile ferroelectric domain wall memory. Sci. Adv..

[40] Shin Y.-H., Grinberg I., Chen I.-W., Rappe A.M. (2007). Nucleation and growth mechanism of ferroelectric domain-wall motion. Nat. Cell Biol..

[41] He D.Y., Qiao L.J., Volinsky A., Bai Y., Guo L.Q. (2011). Electric field and surface charge effects on ferroelectric domain dynamics in BaTiO_3_ single crystal. Phys. Rev. B.

[42] Nelson C.T., Vasudevan R.K., Zhang X.H., Ziatdinov M., Eliseev E.A., Takeuchi I., Morozovska A.N., Kalinin S.V. (2020). Exploring physics of ferroelectric domain walls via Bayesian analysis of atomically resolved STEM data. Nat. Commun..

[43] Seidel J., Martin L.W., He Q., Zhan Q., Chu Y.-H., Rother A., Hawkridge M.E., Maksymovych P., Yu P., Gajek M. (2009). Conduction at domain walls in oxide multiferroics. Nat. Mater..

[44] Guyonnet J., Gaponenko I., Gariglio S., Paruch P. (2011). Conduction at Domain Walls in Insulating Pb(Zr_0.2_Ti_0.8_)O_3_ Thin Films. Adv. Mater..

[45] Lee W., Salje E.K.H., Bismayer U. (2003). Domain-wall structure and domain-wall strain. J. Appl. Phys..

[46] Choudhury S., Li Y., Odagawa N., Vasudevarao A., Tian L., Čapek P., Dierolf V., Morozovska A.N., Eliseev E.A., Kalinin S. (2008). The influence of 180° ferroelectric domain wall width on the threshold field for wall motion. J. Appl. Phys..

[47] Bencan A., Drazic G., Ursic H., Makarovic M., Komelj M., Rojac T. (2020). Domain-wall pinning and defect ordering in BiFeO_3_ probed on the atomic and nanoscale. Nat. Commun..

[48] Wang Y.J., Feng Y.P., Zhu Y.L., Tang Y.L., Yang L.X., Zou M.J., Geng W.R., Han M.J., Guo X.W., Wu B. (2020). Polar meron lattice in strained oxide ferroelectrics. Nat. Mater..

[49] Zuo Y., Genenko Y.A., Klein A., Stein P., Xu B. (2014). Domain wall stability in ferroelectrics with space charges. J. Appl. Phys..

